# Prenatal and Childhood Exposure to Humidifier Disinfectants and Attention-Deficit/Hyperactivity Disorder (ADHD): Insights from a Retrospective Cohort Design

**DOI:** 10.3390/toxics13020078

**Published:** 2025-01-23

**Authors:** Hyowon Choi, Hunju Lee, Yeon-Soon Ahn

**Affiliations:** 1 Department of Prevention Medicine, Yonsei Wonju College of Medicine, Wonju 26426, Republic of Korea; wowo0226@gmail.com (H.C.); hjlee5371@gmail.com (H.L.); 2 Genomic Cohort Institute, Yonsei Wonju College of Medicine, Wonju 26426, Republic of Korea

**Keywords:** attention-deficit disorder with hyperactivity, incidence, environmental exposure, humidifier disinfectants

## Abstract

Humidifier disinfectants (HDs), also known toxic indoor chemicals, have been linked to adverse health outcomes in children. However, their association with ADHD development remains largely unexplored. This study aimed to investigate the incidence of ADHD among HD claimants and assess the association between HD exposure and ADHD risk. A cohort of HD damage claimants born between 2002 and 2011 was established. ADHD cases and controls were identified using matched National Health Insurance Service claim data, with follow-ups required until age 10. ADHD incidence was calculated, and exposure characteristics—including the use of PHMG/PGH, humidifier distance and location, and cumulative and annual exposure duration—were analyzed. Cox proportional hazards analysis was performed, adjusting for sociodemographic factors and comorbidities. Among 1597 subjects, 84 were diagnosed with ADHD, resulting in an incidence density of 4.782 per 1000 person–years. Higher cumulative exposure duration and hours significantly increased the hazard ratio (HR) for ADHD. Additionally, exposure during the first three years after birth was associated with a higher HR for ADHD. These findings suggest that ADHD incidence is elevated in HD claimants and that HD exposure, particularly prolonged or early-life exposure, is associated with increased ADHD risk. Further research is required to confirm these findings in exposed cohorts and to elucidate the mechanisms underlying HD-related ADHD.

## 1. Introduction

Chemicals such as lead, mercury, arsenic, and toluene are known to cause developmental neurotoxicity in humans [1]. Humidifier disinfectants (HDs), indoor toxicants, have been associated with severe lung diseases, including HD-associated lung injury and interstitial lung disease (ILD) [2]. HDs were widely used in Korea, particularly among children and pregnant women. According to Yoon et al., among 1577 randomly selected individuals born in 2008, 75.6% reported using a humidifier, and 31.1% reported using a HD [3]. Despite the widespread use of HDs among children and pregnant women, there is a notable lack of human epidemiological studies addressing the impact of HD exposure on the development of children and adolescents.

Attention-deficit/hyperactivity disorder (ADHD) is the most common neurodevelopmental disorder, characterized by symptoms of inattention, hyperactivity, and impulsivity [4]. The global incidence of ADHD was 307.86 per 100,000 persons in 2019 [5]. ADHD is not caused by a single factor but is often described as the final common pathway resulting from multiple brain developmental processes [6]. Several risk factors have been identified, including genetic factors (e.g., DAT1, DRD4, and DOCK2), psychosocial family stressors, and environmental exposures [7]. Notably, prenatal and early childhood exposure to substances such as fine particulate matter (PM2.5), lead, and tobacco has been identified as a significant risk factor for ADHD [8,9].

In surveys related to HD exposure, ADHD prevalence was remarkably high, with up to 21.4% of children and adolescents reportedly affected [10]. A systematic search was conducted in PubMed in December 2024 using the keywords “Developmental problem” and “humidifier disinfectant”. This search yielded only one relevant study. The study analyzed the association between HD exposure and neuropsychiatric problems in children and adolescents using cross-sectional survey data. Utilizing a screening test, the study reported an adjusted odds ratio (OR) of 1.54 (95% CI: 1.54–2.06) for total neuropsychiatric problems in the HD-exposed group compared to the non-exposed group. For attention-deficit/hyperactivity problems specifically, the adjusted OR was 1.157 (95% CI: 1.03–2.38) [11]. However, this study relied on self-reported data and a screening test, which may introduce inaccuracies in the diagnosis and assessment of HD exposure. Additionally, no studies to date have examined the dose–response relationship or the periodic effects of HD exposure.

This study utilized data from the HD health damage claimant cohort to evaluate the association between HD exposure and ADHD. First, we quantified the magnitude of ADHD by calculating its incidence within the birth cohort of the HD claimants. This approach was designed to indirectly evaluate the potential impact of HD exposure on ADHD risk within this specific population. Second, we assessed the relationship between ADHD and various HD exposure characteristics, including exposure duration, HD type, humidifier location/distance, and specific periodic exposure patterns.

## 2. Materials and Methods

### 2.1. Data Source and Study Populations

From 2011 to 2020, a total of 7056 claimants registered with the Korean Environmental Industry and Technology Institute (KEITI) for relief related to health damages caused by HD. These claimants’ data were systematically documented and stored within KEITI’s comprehensive portal for HD health damage support [12]. We included all individuals born between 2002 and 2011 who were registered in the KEITI database for relief related to HD health damage. A total of 1815 individuals met these criteria and were included in the initial cohort. This study included multiple children from the same family if they met the inclusion criteria, as the analyses focused on individual-level outcomes rather than family-level clustering. This cohort was designated as the Cohort for Survey of Children and Adolescents Affected by HD (CoChAHD). By linking the CoChAHD with the Korean National Health Insurance Service (NHIS) claims data—including outpatient visits, inpatient admissions, procedures, and prescriptions [13]—we tracked medical utilization claims for these subjects from 2002 to 2021, following them up until the age of 10.

**Figure 1:**
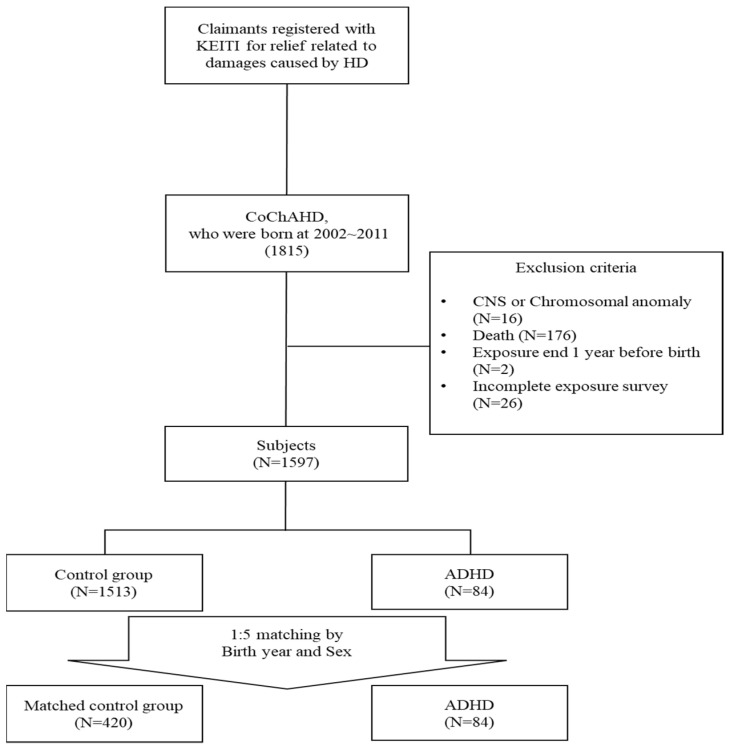
Study flowcharts. KEITI, Korean Environmental Industry and Technology Institute; CoChAHD, Cohort for Survey of Children and Adolescents Affected by Humidifier Disinfectants; CNS, Central Nervous System; ADHD, Attention-Deficit/Hyperactivity Disorder.

Due to the exclusion criteria, deceased subjects (N = 176), those born more than one year after the end of exposure (N = 2), those with incomplete exposure surveys (N = 26), and those with central nervous system or chromosomal anomalies (classified under the International Classification of Diseases, 10th Revision [ICD-10], codes Q0* and Q9*) were excluded. Consequently, a total of 1597 subjects were included in the final analysis (Figure 1).

### 2.2. Outcome of Interest

The principal diagnostic code for ADHD (ICD-10 code F90) was defined as cases where the diagnosis first appeared during the follow-up period, either through outpatient consultations on at least two occasions or inpatient consultations on at least one occasion in a pediatric psychiatry department. Controls were defined as individuals who had no recorded ADHD diagnosis during the follow-up period.

### 2.3. Incidence of ADHD

To estimate the incidence rates (per 1000) of ADHD, we stratified the analysis by sex and two age groups (0–5 years and 6–10 years). The target population was defined as all individuals within the respective age groups and sex categories during the observation period. As the analysis focused on subjects born in 2002, no run-in period was required. Incidence cases were identified as newly diagnosed ADHD cases among subjects observed until the end of the follow-up period. The incidence rate was calculated by dividing the number of incident cases by the target population, multiplied by 1000. Additionally, based on the target population’s birth year, the number of incident cases, and the observation period, we calculated the incidence rate, which accounts for person–years, using Poisson regression.

### 2.4. Assessment of Exposure Characteristics

For variables related to HD exposure, we utilized environmental exposure survey data from KEITI’s comprehensive portal for HD health damage support. The data were obtained through self-administered questionnaires, followed by one-on-one interviews with victims who reported health damage [12]. Subjects’ exposure was verified using HD receipts and photos of HD purchases.

To identify which exposure characteristics were associated with ADHD, we analyzed variables potentially linked to exposure intensity, toxicity, and cumulative effects. Polyhexamethyleneguanidine/oligo(2-(2-ethoxy)ethoxyethyl guanidinium chloride (PHMG/PGH) usage was included because these chemicals are known for their higher toxicity levels and are the most frequently reported HD product types [14]. The distance from the humidifier (“within 1 m” vs. “other”) and the location (“close to nose and mouth” vs. “other”) were chosen to evaluate the intensity of inhalational exposure, as closer proximity is associated with greater respiratory uptake of harmful substances.

To estimate exposure duration, the exposure start age (in months) and exposure end age (in months) were computed based on the date of birth; exposure that occurred before birth was recorded as a negative value. Cumulative exposure duration and cumulative exposure hours were calculated differently for cases and controls to account for their respective characteristics. For the cases, exposure duration was calculated up to the time of ADHD diagnosis to reflect the exposure history relevant to the onset of ADHD. For the controls who did not have an ADHD diagnosis, the cumulative exposure was calculated over the entire follow-up period, up to the age of 10. The cumulative exposure hours were computed by multiplying the years of use, annual use (months/year), monthly use (weeks/month), weekly use (days/week), and daily use (hours/day). These two variables—cumulative exposure duration and cumulative exposure hours—were then categorized into tertiles for analysis.

Prenatal exposure was defined separately for both groups as the number of months of exposure occurring from one year before birth to the birth month. Postnatal exposure was calculated annually until 7 years of age for both groups; however, for the ADHD group, postnatal exposure was calculated only up to the time of ADHD diagnosis.

### 2.5. Covariates

Sociodemographic factors, including sex, birth year, birth season, and rural residence, were included as covariates. Given that humidifier use is prevalent between fall and spring, the birth seasons were categorized as Winter (December to February), Spring (March to May), Summer (June to August), and Fall (September to November). Rural areas were defined according to the Korean residential zoning classification, where regions classified as ’eup’, ’myeon’, or ’ri’ were considered rural.

Asthma (ICD-10 codes J45 and J46), interstitial lung disease (ILD, ICD-10 code J84)—both recognized respiratory conditions associated with HD exposure [15]—and preterm birth (ICD-10 codes P07) were included as comorbidities based on the date of first diagnosis. For the ADHD group, asthma and ILD were defined as conditions diagnosed prior to the diagnosis of ADHD

### 2.6. Statistical Analysis

Data analysis was conducted using R version 4.3.1 (R Foundation for Statistical Computing, Vienna, Austria). Descriptive statistics were generated for all participants, with continuous variables presented as mean ± standard deviation and categorical variables as proportions. A newly developed cohort was extracted from CoChAHD for analysis by matching patients with ADHD to five randomly selected individuals without ADHD, based on their year of birth and sex (Figure 1). Comparative analyses of sociodemographic factors, HD exposure characteristics, and comorbidities between the ADHD and control groups were performed using *t*-tests and chi-square tests, both in unmatched and matched samples.

The Cox Proportional Hazard (PH) model was selected to evaluate the temporal relationship between HD exposure and ADHD occurrence, as it is well-suited for time-to-event data. We performed a Cox PH model, dividing the analysis into two models: Model 1, which was only matched not adjusted, and Model 2, which included additional adjustments for birth season, rural residence, preterm birth, asthma, and ILD. Additionally, we applied landmark analysis to account for time-varying exposure effects. By setting landmarks at tertile values of cumulative exposure and annually for exposure duration, we aimed to capture the dynamic nature of HD exposure and its impact on ADHD risk over time. First, we set a landmark at the end of exposure for tertile values for cumulative exposure duration and hours, distance from a humidifier, location of the humidifier, and usage of PHMG/PGH. In this analysis, we further adjusted for the minimum monthly age at exposure end or at birth. Second, a landmark was set annually to conduct a Cox PH model based on annual exposure duration. This analysis included additional adjustments for exposure status as time-varying components. Statistical significance was determined at *p* < 0.05 for all analyses.

## 3. Results

The characteristics of the study population are as follows: the mean birth year was 2006.7 ± 2.7, the mean age at the start of exposure was 5.3 ± 15.4 months, and the mean total exposure duration was 32.4 ± 26.3 months. The male-to-female ratio was 1.39, and 13.0% of the subjects resided in rural areas. A total of 88.7% of the subjects were ‘PHMG/PGH users’. Additionally, 65.6% of the subjects reported the humidifier distance as “under 1 m”, and 65.6% reported the humidifier’s location as “close to the nose and mouth”. The proportions of asthma and ILD were 83.2% and 6.0%, respectively, while 4.0% of the subjects were born preterm. A total of 84 subjects (5.3%) were in the ADHD group. The mean duration from exposure start to ADHD diagnosis was 85.4 ± 20.2 months, and the mean duration from exposure end to diagnosis was 50.2 ± 35.9 months.

Significant differences were observed in the sex distribution, with a higher proportion of males in the ADHD group (78.6% vs. 57.1%, *p* < 0.001), and in the mean exposure start age (2.8 months for ADHD vs. 5.5 months for controls, *p* = 0.023). Additionally, a higher proportion of preterm births was noted in the ADHD group compared to controls (9.5% vs. 3.7%, *p* = 0.018) (Table 1).

Table 2 illustrates the incidence of ADHD, stratified by sex and age group. The incidence density of ADHD among the study populations was 4.88 per 1000 person–years (95% CI: 3.90–5.96). In males, the incidence density was significantly higher, at 6.63 per 1000 person–years (95% CI: 5.15–8.28), compared to females, who had an incidence density of 2.44 per 1000 person–years (95% CI: 1.45–3.66). Among males, the incidence density in the 6–10-year age group was notably high at 13.33 per 1000 person–years (95% CI: 10.20–16.85), while in females, the highest incidence density was also observed in the 6–10-year age group, at 4.52 per 1000 person–years (95% CI: 2.53–6.99). For the younger age group (0–5 years), males had an incidence density of 1.23 per 1000 person–years (95% CI: 0.50–2.22), and females had an incidence density of 0.74 per 1000 person–years (95% CI: 0.15–1.62).

**Figure 2:**
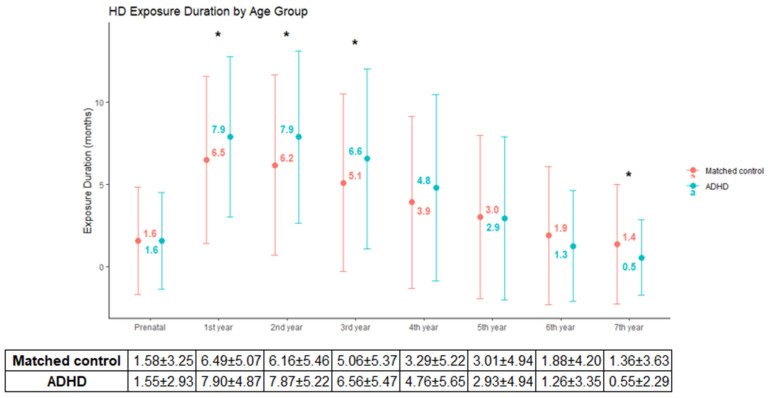
Annual HD exposure duration (months) by ADHD status: Error bars represent standard deviation. Significant differences between groups are marked with asterisks (*).

Table 3 presents the characteristics of the matched study populations, with a total of 504 subjects selected. A significant difference was observed only in the exposure start age in months when compared to the matched control group (*p* < 0.001; Table 2). Figure 2 shows the annual HD exposure duration stratified by ADHD status. Specifically, ADHD subjects experienced higher exposure durations during the early years (1–3 years), with the mean exposure duration in the first year being 7.9 ± 4.9 months for the ADHD group and 6.5 ± 5.1 months for the matched control group (*p* = 0.019). Similarly, exposure durations in the second and third years were 7.9 ± 5.2 and 6.6 ± 5.5 months, respectively, in the ADHD group, compared to 6.2 ± 5.5 and 5.1 ± 5.4 months in the matched control group (*p* = 0.009, *p* = 0.024). However, at 7 years of age, the mean exposure duration was longer in the non-ADHD group (0.6 ± 2.3 months in the ADHD group vs. 1.4 ± 3.6 months in the control group, *p* = 0.007).

Table 4 presents the results of the stratified Cox PH analysis. When analyzed from the end of exposure, the ’PHMG/PGH user status, distance from the humidifier, and location of the humidifier were not statistically significant. However, the third tertile of cumulative exposure duration observed an increased hazard ratio (HR) compared to the first tertile (Model 2, T3 of cumulative exposure duration: HR 2.855, 95% CI 1.163–7.010). In cumulative exposure hours, the second and third tertiles also displayed increased HRs compared to the first tertile (Model 2, T2: HR 1.816, 95% CI 1.003–3.273; Model 2, T3: HR 2.145, 95% CI 1.063–4.326). For annual exposure duration, a significant increase in HR was observed for each additional month of exposure between the first and third years (Model 2, first year: HR 1.058, 95% CI 1.012–1.106; second year: HR 1.062, 95% CI 1.015–1.108; third year: HR 1.050, 95% CI 1.008–1.094). No statistically significant HRs were observed for other years (Table 4).

## 4. Discussion

In this study, using the CoChAHD cohort, we observed a high incidence of ADHD and an association between HD exposure and ADHD development. Specifically, cumulative exposure duration and hours, and longer exposure during the first three years of life were significantly linked to ADHD development.

We conducted the analysis using NHIS claim data. The NHIS claims data covers 97% of the Korean population and includes detailed information on age, sex, residential regions, medical services, and diagnoses, categorized according to ICD-10 [15]. The NHIS claim codes have been consistently utilized since 2002, making it highly likely that most ADHD cases within the CoChAHD cohort were captured in our analysis. However, since the NHIS claim data are administrative claims data, the accuracy of diagnoses may be limited. To enhance the accuracy of ADHD diagnoses in this study, unlike in other studies [16,17], we additionally confirmed either as an outpatient consultation on at least two occasions or as an inpatient consultation on at least one occasion in a pediatric psychiatry department.

In the 2021 Global Burden of Disease Study [18], the annual incidence of ADHD in Korea peaked in 2016 at 264.03 per 100,000 persons in children aged 0–14 years. However, a higher incidence rate was observed in our study population (4.879 per 1000 person-years). In the same study, the Korean incidence rate of ADHD in males was nearly threefold that in females in 2021 (368.1 per 100,000 and 116.61 per 100,000, respectively). Similarly, in our study populations, the incidence density of ADHD in males was nearly threefold that in females (6.628 per 1000 person-years and 2.443 per 1000 person-years, respectively), reflecting a higher incidence compared to the 2021 Global Burden of Disease Study. This higher incidence rate may be attributed to the unique characteristics of our study population, which included a high proportion of individuals with asthma and ILD. Asthma has been strongly associated with ADHD, as demonstrated in a meta-analysis by Kaas et al., which reported an odds ratio (OR) of 1.52 (95% CI: 1.42–1.63, I^2^ = 60%) [19]. Additionally, a study by Giannouli et al. found that adults with ILD (mean age 61.22 ± 12.34 years) exhibited poor neuropsychological function, suggesting that ILD may also impair neurological function [20]. Another potential reason for the higher incidence observed in our study is that the study population consisted of claimants. It is important to note that, prior to the amendment of relevant laws in 2020 [21,22], only respiratory diseases were recognized for claims, excluding ADHD.

The pathological mechanisms of ADHD are not well known. Recent studies have reported that the prefrontal cortex, caudate, and cerebellum are the primary areas of ADHD, and these areas interact with each other to influence attention, thoughts, emotions, behavior, and action [23]. Recent studies have reported that PHMG/PGH and chloromethylisothiazolinone/methylisothiazolinone (CMIT/MIT), the main components of HD, have neurological effects. According to a study by Cho et al., HD causes oxidative stress in zebrafish, which has a toxic effect on the brain. In particular, PHMG affected the midbrain and optic tectum, and CMIT/MIT affected the periventricular gray zone and outer tectal superficial layer [24]. These areas of zebrafish are involved in information processing and behavior control and are known to be involved in the development of nerve cells [24]. In addition, according to Kim and Ji, exposure to 0.15 mg/L of PHMG in zebrafish affected thyroid hormone, reactive oxygen species, and antioxidant enzyme activities, causing developmental retardation [25]. There is a study that reported that benzalkonium chloride, an ingredient used in HD, exhibits neurotoxicity in vitro at concentrations other than cytotoxic concentrations [26]. However, due to the many challenges in applying research findings from zebrafish to humans, further studies in mammals or humans are necessary.

Our study suggested a dose–response relationship in the third tertile of the cumulative exposure duration. The second and third tertiles of cumulative exposure hours represented statistically a significant association with the development of ADHD. Annual HD exposure duration from the first to the third year represented statistically significant associations with ADHD. It is known that the vulnerable period for human brain development is early childhood (the gross brain growth for at least 2~3 years after birth), which is the period when brain development occurs most rapidly [27,28]. Meanwhile, there were no significant results between the prenatal period duration and ADHD. This period is vulnerable to toxins, and exposure to toxins is known to be a risk factor for the development of ADHD [8]. This resulted in a variety of health damage among mothers exposed during that period, of which miscarriages and stillbirths accounted for a large proportion, which led to the formation of an extrapulmonary disease review committee [14].

The strengths of this study include being the first to calculate ADHD incidence within the CoChAHD and to conduct a within-country comparison. This approach provides valuable insights into the impact of HD exposure on ADHD within the same population. Additionally, this study is the first to explore the dose–response relationship and periodic effects of HD exposure on ADHD, using a retrospective cohort design to assess exposure during early childhood.

This study is the first to quantify the incidence of ADHD within the CoChAHD and to demonstrate an association between cumulative HD exposure, longer exposure during the first three years of life—a critical period for brain development—and ADHD risk. However, this study has several limitations. First, we did not have access to key confounding factors such as family history and genetic predisposition, which are known to influence ADHD development. Second, the retrospective collection of HD exposure data, which relied on self-reported use, proximity to the humidifier, and location relative to the mouth and nose, may have introduced information bias due to the lack of environmental or personal monitoring of HD chemicals such as PHMG/PGH. This limitation likely resulted in exposure misclassification, which is expected to be non-differential and would bias the risk estimates toward the null. Furthermore, exposure duration for ADHD cases was calculated only up to the time of ADHD diagnosis, which may not accurately reflect the actual onset of ADHD. This approach, while practical given the available data, could lead to further misclassification of exposure. Third, this study did not involve a direct comparison between exposure and non-exposure groups. Instead, we conducted internal comparisons to minimize selection bias, ensuring that both ADHD cases and controls were drawn from the same population with similar baseline characteristics. However, this approach limited the overall sample size and did not allow for direct comparisons of ADHD incidence between exposure and non-exposure groups, which may have affected the statistical power of the analysis. Finally, as this study employed a retrospective cohort design, it identified an association but did not establish causality.

## 5. Conclusions

Despite these limitations, our findings highlight the potential neurodevelopmental risks associated with HD exposure and underscore the need for further research. Future studies should address confounding factors, involve larger and more diverse cohorts, and focus on mechanistic studies in mammalian models and humans to validate and expand upon these findings.

## Figures and Tables

**Table 1:** Baseline characteristics of the study population.

N (%) or Mean ± SD	ADHD	Control	*p*	Total
	(N = 84)	(N = 1513)		(N = 1597)
Sex			<0.001	
M	66 (78.6%)	864 (57.1%)		930 (58.2%)
F	18 (21.4%)	649 (42.9%)		667 (41.8%)
Birth year	2006.4 ± 2.8	2006.7 ± 2.7	0.397	2006.7 ± 2.7
Urbanization			0.258	
Other place	77 (91.7%)	1313 (86.8%)		1390 (87.0%)
Rural area	7 (8.3%)	200 (13.2%)		207 (13.0%)
Birth season			0.069	
Winter (December~February)	17 (20.2%)	406 (26.8%)		378 (23.7%)
Spring (March~May)	20 (23.8%)	371 (24.5%)		391 (24.5%)
Summer (June~Auguest)	14 (16.7%)	375 (24.8%)		389 (24.4%)
Fall (September~November)	33 (39.3%)	361 (23.9%)		439 (27.5%)
Exposure start age at month	2.8 ± 10.0	5.5 ± 15.7	0.023	5.3 ± 15.4
Exposure end age at month	38.6 ± 27.1	37.7 ± 28.1	0.773	37.7 ± 28.1
Antenatal exposure duration	1.6 ± 2.9	1.9 ± 3.8	0.382	1.8 ± 3.7
Cumulative exposure duration (before diagnosis ADHD), month	33.5 ± 23.8	32.2 ± 26.2	0.651	32.3 ± 26.1
Cumulative exposure duration (before diagnosis ADHD), Tertile			0.595	
-T1 (≥1, <16)	26 (31.0%)	528 (34.9%)		554 (34.7%)
-T2 (≥16, <39)	27 (32.1%)	504 (33.3%)		531 (33.2%)
-T3 (≥39, <145)	31 (36.9%)	481 (31.8%)		512 (32.1%)
Cumulative exposure hours (before diagnosis ADHD)	14,391.9 ± 14,078.7	12,265.8 ± 12,521.6	0.133	12,377.6 ± 12,612.3
Cumulative exposure hours (before diagnosis ADHD), Tertile			0.231	
-T1 (≥48, <4704)	21 (25.0%)	515 (34.0%)		536 (33.6%)
-T2 (≥4704, <13,244)	31 (36.9%)	499 (33.0%)		530 (33.2%)
-T3 (≥13,244, <91,392)	32 (38.1%)	499 (33.0%)		531 (33.2%)
Usage of PHMG/PGH			0.471	
N	12 (14.3%)	168 (11.1%)		180 (11.3%)
Y	72 (85.7%)	1345 (88.9%)		1417 (88.7%)
Distance to humidifier			0.296	
under 1 m	60 (71.4%)	987 (65.2%)		1047 (65.6%)
over 1 m	24 (28.6%)	526 (34.8%)		550 (34.4%)
Location of humidifier			0.736	
Close to nose and mouth	57 (67.9%)	990 (65.4%)		1047 (65.6%)
Other locations	27 (32.1%)	523 (34.6%)		550 (34.4%)
Interstitial lung disease			0.104	
N	75 (89.3%)	1426 (94.2%)		1501 (94.0%)
Y	9 (10.7%)	87 (5.8%)		96 (6.0%)
Asthma			0.471	
N	17 (20.2%)	251 (16.6%)		268 (16.8%)
Y	67 (79.8%)	1262 (83.4%)		1329 (83.2%)
Preterm birth			0.018	
N	76 (90.5%)	1457 (96.3%)		1533 (96.0%)
Y	8 (9.5%)	56 (3.7%)		64 (4.0%)

ADHD, Attention-deficit/hyperactivity disorder; PHMG/PGH, polyhexamethyleneguanidine/oligo(2-(2-ethoxy)ethoxyethyl guanidium chloride.

**Table 2:** Incidence of ADHD in study populations.

Incidence (/1000 PY)	Male		Female		Total
	Incidents	Incidence (95%CI)	Incidents	Incidence (95%CI)	Incidence (95%CI)
0–5 years-old	7	1.23 (0.50–2.22)	3	0.74 (0.15–1.62)	1.03 (0.49–1.72)
6–10 years-old	61	13.33 (10.20–16.85)	15	4.52 (2.53–6.99)	9.63 (7.58–11.89)
Total	68	6.63 (5.15–8.28)	18	2.44 (1.45–3.66)	4.88 (3.90–5.96)

**Table 3:** Matched data characteristics of ADHD and control groups.

N (%) or Mean ± SD	ADHD	Matched Control	*p*	Matched Total
	(N = 84)	(N = 420)		(N = 504)
Sex			1	
M	66 (78.6%)	330 (78.6%)		396 (78.6%)
F	18 (21.4%)	90 (21.4%)		108 (21.4%)
Birth year	2006.4 ± 2.8	2006.4 ± 2.8	1	2006.4 ± 2.8
Urbanization			0.417	
Other place	77 (91.7%)	369 (87.9%)		446 (88.5%)
Rural area	7 (8.3%)	51 (12.1%)		58 (11.5%)
Birth season			0.127	
Winter (December~February)	17 (20.2%)	109 (26.0%)		126 (25.0%)
Spring (March~May)	20 (23.8%)	98 (23.3%)		118 (23.4%)
Summer (June~Auguest)	14 (16.7%)	98 (23.3%)		112 (22.2%)
Fall (September~November)	33 (39.3%)	115 (27.4%)		148 (29.4%)
Exposure start age at month	2.8 ± 10.0	7.7 ± 17.3	<0.001	6.9 ± 16.4
Exposure end age at month	38.6 ± 27.1	38.6 ± 28.7	0.994	38.6 ± 28.4
Antenatal exposure duration	1.6 ± 2.9	1.6 ± 3.3	0.955	1.6 ± 3.2
Cumulative exposure duration (before diagnosis ADHD), month	33.5 ± 23.8	30.8 ± 25.8	0.374	31.3 ± 25.4
Cumulative exposure duration (before diagnosis ADHD), Tertile			0.366	
-T1 (≥1, <16)	26 (31.0%)	156 (37.1%)		182 (36.1%)
-T2 (≥16, <39)	27 (32.1%)	140 (33.3%)		167 (33.1%)
-T3 (≥39, <145)	31 (36.9%)	124 (29.5%)		155 (30.8%)
Cumulative exposure hours (before diagnosis ADHD)	14,391.9 ± 14,078.7	11,189.1 ± 11,149.4	0.052	11,722.9 ± 11,733.9
Cumulative exposure hours (before diagnosis ADHD), Tertile			0.147	
-T1 (≥48, <4704)	21 (25.0%)	151 (36.0%)		172 (34.1%)
-T2 (≥4704, <13,244)	31 (36.9%)	138 (32.9%)		169 (33.5%)
-T3 (≥13,244, <91,392)	32 (38.1%)	131 (31.2%)		163 (32.3%)
Usage of PHMG/PGH			0.371	
N	12 (14.3%)	43 (10.2%)		55 (10.9%)
Y	72 (85.7%)	377 (89.8%)		449 (89.1%)
Distance to humidifier			0.099	
under 1 m	60 (71.4%)	257 (61.2%)		317 (62.9%)
over 1 m	24 (28.6%)	163 (38.8%)		187 (37.1%)
Location of humidifier			0.617	
Close to nose and mouth	57 (67.9%)	270 (64.3%)		327 (64.9%)
Other locations	27 (32.1%)	150 (35.7%)		177 (35.1%)
Interstitial lung disease			0.517	
N	75 (89.3%)	387 (92.1%)		462 (91.7%)
Y	9 (10.7%)	33 (7.9%)		42 (8.3%)
Asthma			0.601	
N	17 (20.2%)	72 (17.1%)		89 (17.7%)
Y	67 (79.8%)	348 (82.9%)		49.7 ± 36.0
Preterm birth			0.246	
N	76 (90.5%)	397 (94.5%)		473 (93.8%)
Y	8 (9.5%)	23 (5.5%)		31 (6.2%)

ADHD, Attention-deficit/hyperactivity disorder; PHMG/PGH, polyhexamethyleneguanidine/oligo(2-(2-ethoxy)ethoxyethyl guanidium chloride.

**Table 4:** Cox Proportional Hazard regression analysis for ADHD risk factors.

Variables	Model1 HR (95%CI)	Model2 HR (95%CI)
From the end of exposure		
Usage of PHMG/PGH,		
Non PHMG/PGH	1.00 (reference)	1.00 (reference)
PHMG/PGH user	0.67 (0.36–1.24)	0.70 (0.37–1.31)
Distance to humidifier		
Under 1 m	1.00 (reference)	1.00 (reference)
Over 1 m	0.63 (0.38–1.04)	0.64 (0.39–1.06)
Location of humidifier		
close to nose and mouth	1.00 (reference)	1.00 (reference)
Others	0.89 (0.55–1.44)	0.94 (0.58–1.52)
Cumulative exposure duration (before diagnosis ADHD), Tertile		
-T1 (≥1, <16)	1.00 (reference)	1.00 (reference)
-T2 (≥16, <39)	1.12 (0.65–1.94)	1.43 (0.77–2.64)
-T3 (≥39, <145)	1.48 (0.85–2.59)	2.86 (1.167–7.01)
Cumulative exposure hours (before diagnosis ADHD), Tertile		
-T1 (≥48, <4704)	1.00 (reference)	1.00 (reference)
-T2 (≥4704, <13,244)	1.56 (0.88–2.76)	1.82 (1.01–3.27)
-T3 (≥13,244, <91,392)	1.73 (0.97–3.10)	2.15 (1.06–4.33)
Annual exposure duration		
Prenatal period	1.00 (0.93–1.07)	1.00 (0.93–1.07)
1st year	1.06 (1.01–1.11)	1.06 (1.01–1.11)
2nd year	1.06 (1.02–1.10)	1.06 (1.02–1.11)
3rd year	1.05 (1.01–1.09)	1.05 (1.01–1.09)
4th year	1.03 (0.99–1.07)	1.03 (0.99–1.08)
5th year	1.00 (0.96–1.05)	1.01 (0.96–1.06)
6th year	0.98 (0.92–1.04)	0.99 (0.91–1.06)
7th year	0.92 (0.82–1.03)	0.92 (0.80–1.06)

PHMG/PGH, polyhexamethyleneguanidine/oligo(2-(2-ethoxy)ethoxyethyl guanidium chloride HR, hazard ratio; CI, confidential index; Model 1, matched; and Model 2: adjusted for matching variables plus additional covariates (birth season, rural residence, preterm birth, asthma, and ILD). For end-of-exposure analyses, monthly age at the end of exposure was included as an additional covariate, while for annual exposure duration analyses, exposure status was treated as a time-varying component.

## Data Availability

The datasets presented in this article are not readily available due to the privacy of the participants. Requests to access the datasets should be directed to Y.-S.A.
